# Effects of Exercise Intervention on Mitochondrial Stress Biomarkers in Metabolic Syndrome Patients: A Randomized Controlled Trial

**DOI:** 10.3390/ijerph18052242

**Published:** 2021-02-24

**Authors:** Jae Seung Chang, Jun Namkung

**Affiliations:** 1 Mitohormesis Research Center, Yonsei University Wonju College of Medicine, 20 Ilsan-ro, Wonju, Gangwon-do 26426, Korea; godbless@yonsei.ac.kr; 2 Yonsei Institute of Sports Science & Exercise Medicine (YISSEM), 20 Ilsan-ro, Wonju, Gangwon-do 26426, Korea; 3 Department of Biochemistry, Yonsei University Wonju College of Medicine, 20 Ilsan-ro, Wonju, Gangwon-do 26426, Korea

**Keywords:** metabolic syndrome, exercise training, randomized controlled trial, biomarker, growth differentiation factor 15, fibroblast growth factor 21, angiopoietin-like 6, leptin

## Abstract

Metabolic syndrome (MetS) pathogenesis involves oxidative stress associated with mitochondrial dysfunction, which triggers integrated stress responses via various compensatory metabolic modulators like mitokines and hepatokines. However, the regulatory mechanisms underlying the exercise-derived benefits with respect to mitokines and hepatokines (potential MetS biomarkers) are unknown. Thus, we investigated the effects of exercise training on MetS biomarkers and their associations with clinical parameters. In this single-center trial, 30 women with MetS were randomly assigned to 12-week supervised exercise or control groups (1:1) and compared with 12 age-matched healthy volunteers. All participants completed the study except one subject in the control group. Expectedly, serum levels of the mitokines, fibroblast growth factor-21 (FGF21), growth differentiation factor-15 (GDF15), and the hepatokine, angiopoietin-like 6 (ANGPTL6), were higher in MetS patients than in healthy volunteers. Moreover, their levels were markedly attenuated in the exercise group. Further, exercise-mediated changes in serum FGF21 and GDF15 correlated with changes in the homeostasis model of assessment of insulin resistance (HOMA-IR) and appendicular lean mass (ALM), respectively. Additionally, changes in serum triglycerides and ANGPTL6 were correlated with changes in leptin. Aberrant mitokine and hepatokine levels can be rectified by relieving metabolic stress burden. Therefore, exercise training may reduce the need for the compensatory upregulation of MetS metabolic modulators by improving gluco-lipid metabolism.

## 1. Introduction

Metabolic syndrome (MetS) refers to a cluster of interrelated risk factors for type 2 diabetes (T2DM) and atherosclerotic and non-atherosclerotic cardiovascular diseases (CVD) [1,2,3]. These include central obesity, impaired glucose homeostasis, dyslipidemia, and elevated blood pressure [4]. The pathogenesis of MetS involves metabolic stress and mitochondrial dysfunction associated with obesity and insulin resistance [5,6]. Chronic overnutrition and physical inactivity result in an accumulation of fat in the muscle, liver, and adipose tissues and lead to insulin resistance [7,8], causing the production of reactive oxygen species (ROS) in the cells. Subsequently, this oxidative stress further aggravates ROS overproduction from mitochondria and endoplasmic reticulum (ER), resulting in serious mitochondrial damage and ER stress.

Mitochondria play a crucial role in cellular energy metabolism and act as an important mediator in adaptive compensatory response to cellular stresses. The adaptive responses to metabolic stress encompass upregulated mitochondrial biogenesis, altered bioenergetic status, and enhanced antioxidant defense system. These integrated stress responses (ISR) improve metabolic and mitochondrial flexibilities, consequently contributing to the amelioration or delay of the onset of metabolic diseases. ISR may be elicited by mitokines which are cell autonomous and non-autonomous signals induced by mitochondrial stress. Fibroblast growth factor 21 (FGF21) and growth differentiation factor 15 (GDF15), prominently expressed in liver and muscle tissues involved in glucose and lipid metabolism, are notably upregulated and secreted following mitochondrial stress and disorders [9,10,11,12]. Indeed, these mitokines play protective roles against mitochondrial injury and metabolic exacerbation [13,14]. High circulating levels of FGF21 and GDF15 are induced upon exposure to sustained and uncompensated metabolic stress to restore metabolic homeostasis. Thus, serum mitokine levels may prove to be potential biomarkers reflecting the extent of metabolic disturbance attributable to mitochondrial stresses.

Among several insulin-sensitive tissues susceptible to metabolic disorders, liver is evidently recognized as an endocrine organ and target of metabolic fitness that releases hepatokines. Angiopoietin-like 6 (ANGPTL6) is a hepatokine that has the potential to enhance insulin sensitivity, increase energy expenditure, and protect against diet-induced obesity [15]. Despite its beneficial effects on metabolic profiles, clinical studies have consistently shown paradoxical upregulation of circulating ANGPTL6 in patients with metabolic disorders [16,17]; thus, similar to the mitokines FGF21 and GDF15, ANGPTL6 may also be considered a candidate biomarker reflecting a compensatory mechanism against metabolic stress.

Exercise is one of the most potent lifestyle modalities that prevents and mitigates chronic metabolic diseases such as MetS, T2DM, and cardiovascular diseases [18]. Enhanced physical fitness and improved body composition through exercise training may contribute to the alleviation of metabolic and oxidative stresses, leading to the restoration of the aberrant serum levels of the relevant biomarkers. Although some reports on the biomarker level alterations occurring in response to acute exercise regimens or in adaptation to exercise training exist, the effect of exercise in metabolic disorders patients remains unclear. Therefore, in this study, we aimed to examine the changes in the serum levels of FGF21, GDF15, and ANGPTL6 elicited by long-term exercise intervention in MetS patients. Our preliminary observations comprised an age-matched comparison of baseline levels between MetS patients and healthy individuals. In addition, we assessed the effects of exercise intervention on kinanthropometric parameters and blood metabolic profiles and analyzed their relationship with the metabolic stress biomarkers. Here, we show that exercise intervention helped decrease the levels of these modulators of metabolic stress in addition to improving physical fitness in MetS patients.

## 2. Materials and Methods

### 2.1. Study Design and Ethical Approval

This study is a single-center randomized controlled trial and was registered at the International Clinical Trial Registry Platform of the World Health Organization (KCT 0004951). The Institutional Review Board of Wonju Christian Hospital approved the study design (IRB No. CR316057) and all participants provided written informed consent. The study was performed in accordance with the principle of the Declaration of Helsinki and the guideline of Consolidated Standards of Reporting Trials (CONSORT) (Table S1). 

### 2.2. Participants

#### 2.2.1. Patients with Metabolic Syndrome

Women previously diagnosed with MetS via health examination were recruited from February to June 2019 at the Center for Exercise Medicine (Yonsei University Wonju College of Medicine, Gangwon-do, Korea). Thirty women with MetS were initially enrolled, but twenty-nine subjects completed the present intervention. MetS was defined according to the criteria of the International Diabetes Federation [4]: central obesity (waist circumference ≥ 80 cm) and the presence of at least 2 of the following components: (1) serum triglycerides ≥ 150 mg/dL or specific treatment for hypertriglyceridemia, (2) serum high-density lipoprotein (HDL) cholesterol < 50 mg/dL or specific treatment for hypo-HDL cholesterolemia, (3) systolic blood pressure (BP) ≥ 130 mmHg or diastolic BP ≥ 85 mmHg or treatment for previously diagnosed hypertension, and (4) fasting plasma glucose ≥ 100 mg/dL or previously diagnosed as T2DM. Women who (i) had participated in regular exercise or sports activity more than 2 times per week within the last 3 months, (ii) had exercise intolerance due to various medical conditions including cardiovascular and musculoskeletal injury, or (iii) did not wish to continue participating in the randomly assigned intervention program, were excluded from the study.

#### 2.2.2. Age-Matched Healthy Volunteers

For comparison of baseline data of the primary outcome between MetS patients and healthy persons, 12 age-matched metabolically healthy women volunteers were recruited in the initial phase of the present study.

### 2.3. Randomization and Blinding

After pre-intervention assessment, 30 MetS women were randomly assigned 1:1 into either an exercise training or a usual care control group using a computer-generated random number table with sealed envelopes. Outcome measurements were conducted by research technicians blinded to subject allocation. The study statistician was also unaware of the group allocations until after data analysis.

### 2.4. Intervention Study

#### 2.4.1. Exercise Intervention Group

The exercise training program was designed using guidelines on optimal exercise intensity, frequency, time, and type given in the American College of Sports Medicine’s Exercise Management for Persons with Chronic Diseases and Disabilities [19]. To maximize the physical and metabolic benefits from exercise training, we adopted a combined endurance and strength exercise regimen. We used a 12-week intervention program which enhanced physical fitness, as well as metabolic improvements were shown [20,21]. Participants performed a 50 min exercise routine that combined step aerobics using height-adjustable platforms and progressive strength training using various body weights and elastic bands (Thera-Band, Hygenic Co., Akron, OH, USA). Each exercise session began with a warm-up, consisting of 5 min stretching and bare gymnastics, and finished with a cool-down comprising of 5 min mild to moderate walking and stretching of the exercised muscles. The aerobic exercise training was conducted at the beginning with individual’s ratings of perceived exertion (RPE) intensities within 11–13 and was progressively encouraged at every 4-week interval until reaching a rating of 14–16. The aerobic exercises consisted of basic step-ups (front, side, lateral, multi-directional), modified step-ups and side steps with knee raise, low kicks, hop and jack, or row and lift, side leg swings, crossovers on step, alternative toe touches, and jack exercises (step/side/march/jumping). The resistance exercises incorporated shoulder press/pull-down, Blackburn, push-up, biceps curl, triceps kickback, abdominal curl-up, plank, squat, lunge, and calf raise, and participants were encouraged to finally complete three sets for each training session of 10–12 repetitions to volitional fatigue. The elastic-resistance of Thera-band was determined by the OMNI Resistance Exercise Scale (OMNI-RES) ratings of perceived exertion and the 2 for 2 rule for increasing training loads [22,23]. The elastic resistance training was conducted within ranges from 7 to 8 on the OMNI-RES. If subjects could complete more than 13 repetitions in the last session and appeared to be lower than 6 (somewhat hard) on the OMNI-RES, the elastic-resistance of her band was increased to the next level. The exercises using body-weight were progressively made more challenging by adjusting the repetition and set numbers performed for each exercise in line with each subject’s improvement. To raise training attendance rate, the exercise classes that consisted of the same programs under the instruction of an exercise specialist were held twice every weekday, and the participants were encouraged to attend either of the two classes more than three days weekly during the 12-week intervention period.

#### 2.4.2. Usual Care Control Group

Subjects in the usual-care control group were instructed to sustain their routine lifestyle including leisure and occupational physical activities, but not to take part in any new physical training programs for 12 weeks. Post-study, the control subjects, who undertook both pre- and post-intervention assessments, were provided the opportunity of participating in the same exercise programs as the exercise intervention group.

### 2.5. Outcome Measures

The primary outcome measured was serum levels of FGF21, GDF15, and ANGPTL6. The secondary outcome measured included kinanthropometric profiles (i.e., anthropometric and body composition indices, physical fitness parameters) and routine biochemical markers in patients with metabolic disorders. All measurements were obtained at baseline and after 12 weeks, at the end of the study period.

#### 2.5.1. Blood Collection and Biochemical Measurements

Blood samples after overnight fasting (≥10 h) were drawn from the antecubital vein for analyses for biochemical parameters and novel biomarkers of metabolic diseases. The separated sera were immediately stored at −80 °C until assay. Baseline and post-intervention blood samples were collected at least 2 days before the intervention and 2 days after the last exercise session, respectively. Serum concentrations of biomarkers were quantified by the enzyme-linked immunosorbent assay (ELISA) using commercial kits targeting FGF21 (DF2100), GDF15 (DGD150), leptin (DLP00) (R&D System, Minneapolis, MN, USA), and ANGPTL6 (AG-45A-0016Y) (Adipogen, Seoul, Korea) according to the manufacturer’s instructions. Biochemical tests for gluco-lipid profiles and hepatocellular enzymes in serum were performed using an automated clinical chemistry analyzer (Roche Cobas^®^ 8000) with the manufacturer’s reagents and calibrators (Roche, Mannheim, Germany). The homeostasis model of assessment of insulin resistance (HOMA-IR) was calculated using the following formula: (fasting insulin (μU/mL) × fasting glucose (mg/dL))/405 and (360 × fasting insulin (μU/mL))/(fasting glucose (mmol/L) – 63), respectively [24].

#### 2.5.2. Kinanthropometric Profiles

Body weight and height were measured in duplicates and used to calculate body mass index (BMI). Waist circumference was measured at the midway between the lowest rib and the superior border of the iliac crest. Body composition variables including body fat mass and percentage, and appendicular lean mass (ALM) were measured using a multi-frequency bioelectrical impedance analyzer (Inbody 720, Biospace, Seoul, Korea). Subjects were evaluated for health-related components of physical fitness such as muscular power, strength, endurance, agility, aerobic capacity, and flexibility through the assessments of standing long jump, handgrip strength, sit-ups, 10 m shuttle run, 20 m pacer, and sit-and-reach tests, respectively.

### 2.6. Statistical Analysis

Using a sample size calculator (Available online: https://clincalc.com/stats/samplesize.aspx (accessed on 27 July 2019)) with anticipated mean and standard deviation (SD) of GDF15 levels in the baseline of 893.6 ± 252.3 pg/mL [25], anticipated percent difference in outcomes of exercise group of 30%, type 1 error rate of 5%, and power of 80%, we found that a sample size of at least 14 subjects per study arm was needed in order to detect the change between baseline and after intervention in both groups. Therefore, we found that 30 subjects were enough for statistical analyses. Data were analyzed using SPSS 25.0 software (IBM Corp., Armonk, NY, USA). A 2-sided *p*-value of <0.05 was considered statistically significant. Continuous data were tested for normality using Shapiro–Wilk tests and are presented as mean ± standard deviation (for parametric variables) unless otherwise noted, or median with interquartile ranges (for non-parametric variables). Categorical data were expressed as frequencies with proportion. Comparisons of continuous variables between groups were conducted using the independent *t*-test or Mann–Whitney *U* test, whereas within-group changes from baseline to 12 weeks were assessed using the paired *t*-test or Wilcoxon signed-rank tests, as appropriate. Categorical variables were analyzed using the Fisher’s exact test. The relationships between the metabolism-related biomarkers and other clinical parameters were assessed by Spearman’s correlation coefficient (r). All graphs were generated using GraphPad Prism 6.0 software (GraphPad Software, Inc., San Diego, CA, USA).

## 3. Results

### 3.1. MetS Women Have Higher Serum FGF21, GDF15, and ANGPTL6 Levels Than Age-Matched Healthy Women

**Figure 1:**
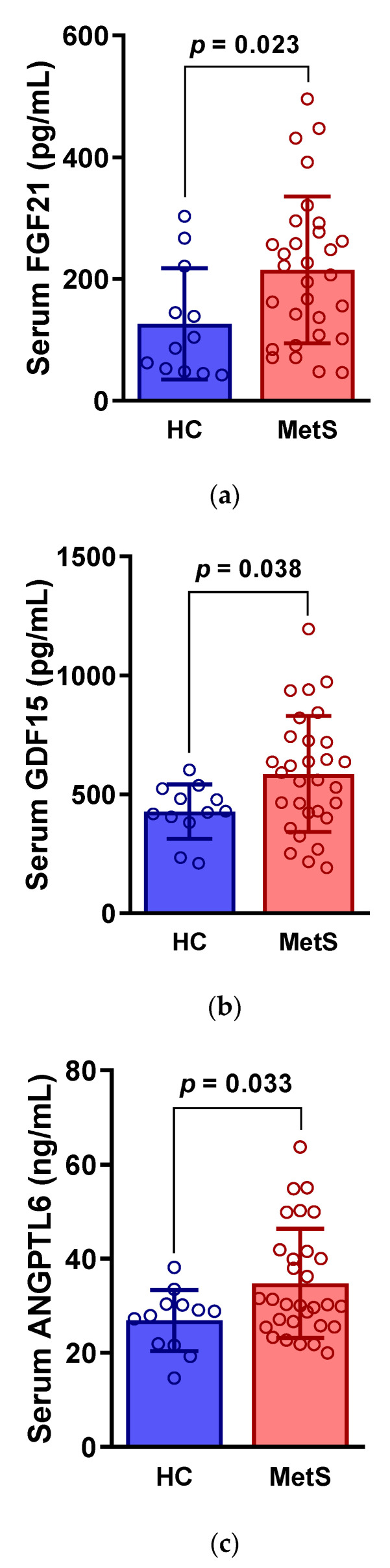
Serum levels of (**a**) fibroblast growth factor 21 (FGF21), (**b**) growth differentiation factor 15 (GDF15), and (**c**) angiopoietin-like 6 (ANGPTL6) between metabolic syndrome (MetS) patients and age-matched healthy controls (HC). Circles are individual values.

Independent *t*-test indicated no significant difference in mean age (mean ± SD; 58.3 ± 6.0 vs. 59.4 ± 10.3 years, *p* = 0.54) between MetS patients and healthy controls. The age-matched healthy women had similar ALM and muscular strength as the MetS subjects (Appendix A Table A1). MetS women showed significantly higher serum levels of FGF21 (215.0 ± 120.6 vs. 126.2 ± 91.5 pg/mL) and GDF15 (585.3 ± 243.7 vs. 427.0 ± 114.6 pg/mL) than the age-matched healthy women. In addition, serum ANGPTL6 levels were also significantly higher in MetS women than in healthy controls (34.7 ± 11.6 vs. 26.9 ± 6.5 ng/mL) (Figure 1).

### 3.2. Characteristics and Disposition of the Participants with Metabolic Syndrome

**Figure 2:**
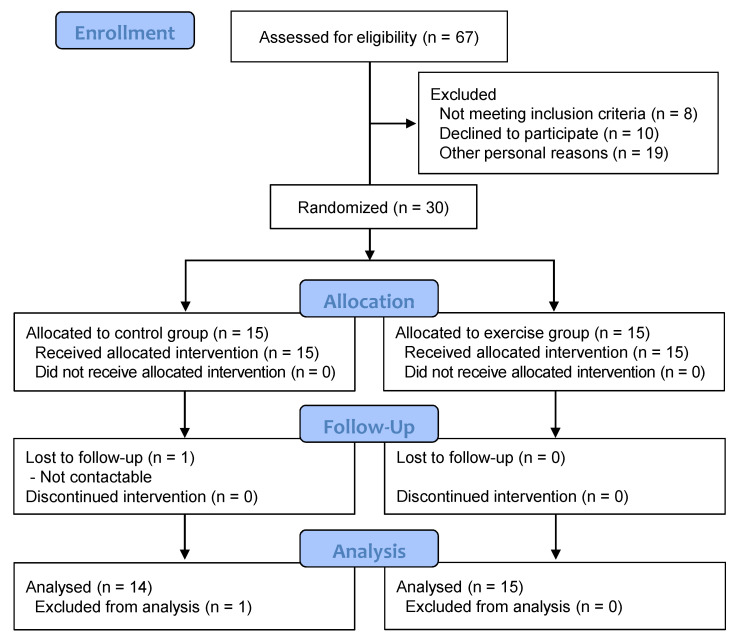
CONSORT flow chart of the exercise intervention study.

A CONSORT diagram of participation throughout the study is detailed in Figure 2. Of the 67 participants, 30 eligible MetS patients were randomly divided equally between control and exercise groups. All subjects in the exercise group completed the recommended exercise training program with an average attendance rate of 93% (range 82–100%) for all sessions. However, one subject in the control group was excluded from the final analysis because she remained uncontactable during follow-up measurements. Baseline demographic and clinical characteristics of the randomized MetS patients are shown in Table 1 and Table 2. There were no baseline differences between the control and exercise groups for each component of MetS. In addition, no statistically significant differences in body composition, physical fitness variables, and blood biochemical parameters were found between the groups.

### 3.3. Effect of Exercise Intervention on the Study Outcomes

#### 3.3.1. Primary Outcomes: Exercise Suppresses Serum FGF21, GDF15, and ANGPTL6 Levels in MetS Women

**Figure 3:**
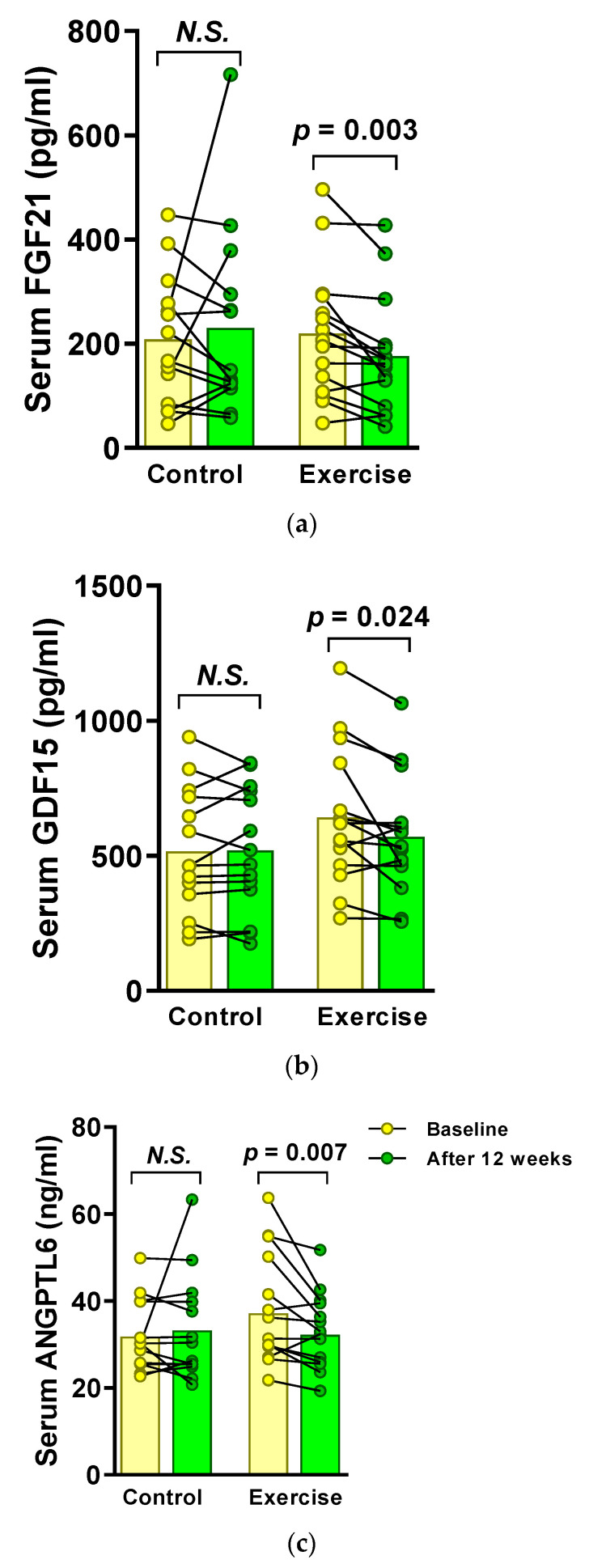
Individual and mean changes in circulating levels of (**a**) fibroblast growth factor 21 (FGF 21), (**b**) growth differentiation factor 15 (GDF15), and (**c**) angiopoietin-like 6 (ANGPTL6) elicited by a 12-week exercise training in metabolic syndrome patients. Circles and bars represent individual data and the means, respectively. *p*-values were determined by Wilcoxon signed-rank test (for FGF21 and ANGPTL6) or Student’s paired *t*-test (for GDF15). N.S., not statistically significant.

Individual and mean changes in serum FGF21, GDF15, and ANGPTL6 levels are shown in Figure 3. A 12-week exercise intervention elicited noteworthy reductions in the serum levels of FGF21 (baseline vs. after 12 weeks, mean ± standard error of the mean (SEM), 219.7 ± 32.2 vs. 176.3 ± 28.7 pg/mL) and GDF15 (642.6 ± 65.0 vs. 570.3 ± 56.1 pg/mL), whereas no changes were detected in the control group (FGF21: 208.2 ± 33.3 vs. 230.2 ± 48.4 pg/mL, *p* = 0.625; GDF15: 516.3 ± 62.3 vs. 520.5 ± 61.9 pg/mL, *p* = 0.829). Similarly, serum ANGPTL6 levels were also reduced in the exercise group (37.2 ± 3.4 vs. 32.2 ± 2.3 ng/mL), but no significant change occurred in the control group (31.8 ± 2.2 vs. 33.2 ± 3.2 ng/mL, *p* = 0.807).

#### 3.3.2. Improved Kinanthropometric Parameters in Exercising MetS Women

The 12-week exercise training resulted in noticeable enhancement of the kinanthropometric parameters, as shown in Table 1. The exercise group, but not the control group, displayed significant 6.2% and 6.5% reductions in waist circumference and body fat percentage respectively, with a 4.6% increase in the ALM/BFM ratio, which represents relative muscle mass and is predictive of MetS development [26]. Likewise, significant improvements in the health-related physical fitness components were observed only in exercise-trained MetS women. Moreover, sit-up and handheld dynamometer tests showed that exercise training significantly enhanced muscular endurance and strength (40.9% and 5.1% enhancement, respectively). Furthermore, the 20 m pacer and the sit-and-reach tests revealed that aerobic capacity and flexibility increased by 33.0% and 15.8%, respectively. However, agility and muscular power remained unaltered during the intervention period.

#### 3.3.3. Blood Biochemical Profiles and the Number of Metabolic Syndrome Components

The number of MetS components was noticeably decreased after exercise training, whereas the change in the non-exercise control group was not significant (Table 2). Serum leptin levels were also reduced in the exercise group (baseline vs. after 12 weeks, mean ± SEM, 8.7 ± 1.1 vs. 7.3 ± 1.2 ng/mL, *p* = 0.041), but no significant change was observed in the control group (12.1 ± 2.0 vs. 12.5 ± 1.9 ng/mL, *p* = 0.433). Post-intervention, a modest decrease from the baseline level of glycated hemoglobin A1c was observed in the exercise group (*p* = 0.029). Although not significant, exercise intervention had a dampening effect on biochemical variables such as triglyceride levels (*p* = 0.069), γ-glutamyl transferase levels (*p* = 0.064), HOMA-IR (*p* = 0.084), and high-sensitivity C-reactive protein levels (*p* = 0.067), which reflected metabolic disorders; contrastingly, HDL-cholesterol levels seemed to increase (*p* = 0.078).

#### 3.3.4. Relationship between Changes in Metabolism-Related Humoral Factors and Clinical Parameters

**Figure 4:**
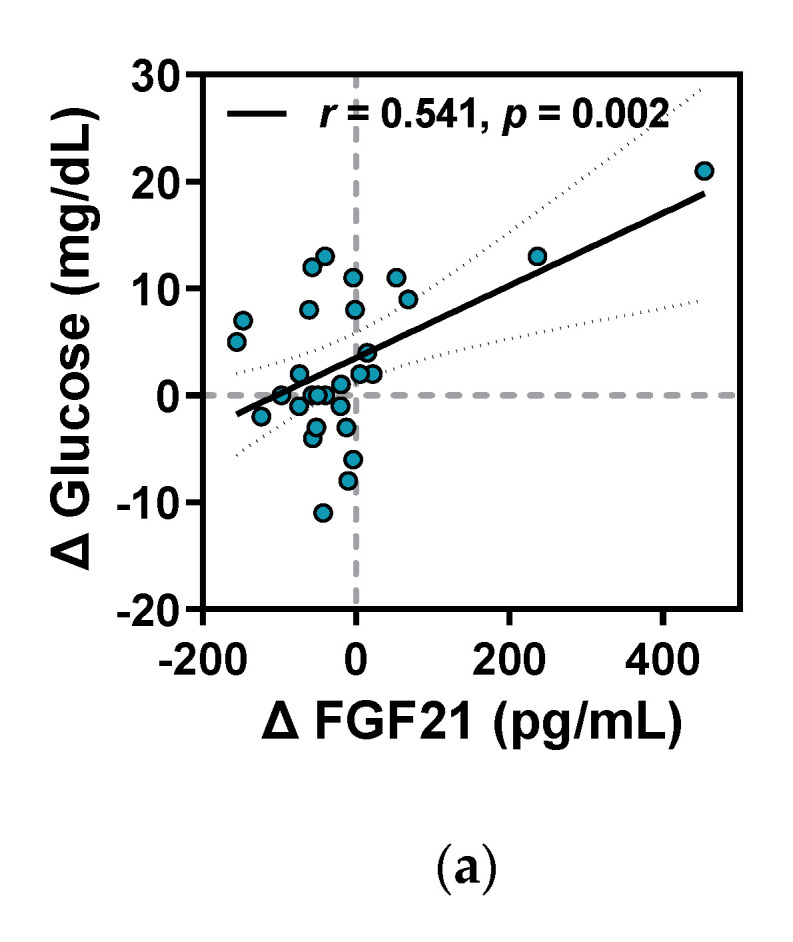

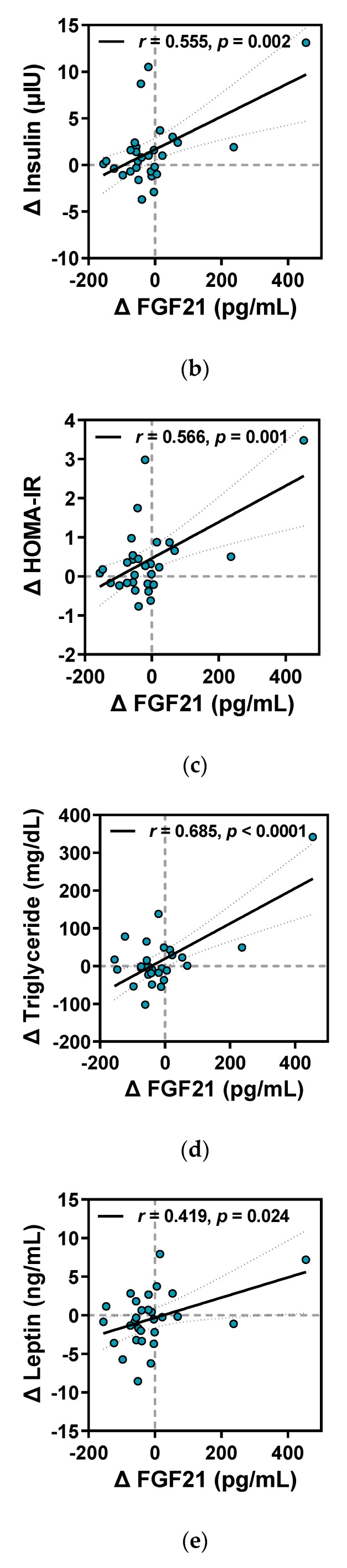
Correlations of Δ FGF21 level with (**a**) Δ glucose, (**b**) Δ insulin, (**c**) Δ HOMA-IR, (**d**) Δ triglyceride, and (**e**) Δ leptin levels before and after the intervention. Correlation coefficient (r), corresponding regression and 95% confidence interval (CI) lines, and *p*-values are represented in the respective panels. Blue-filled circles are individual values.

**Figure 5:**
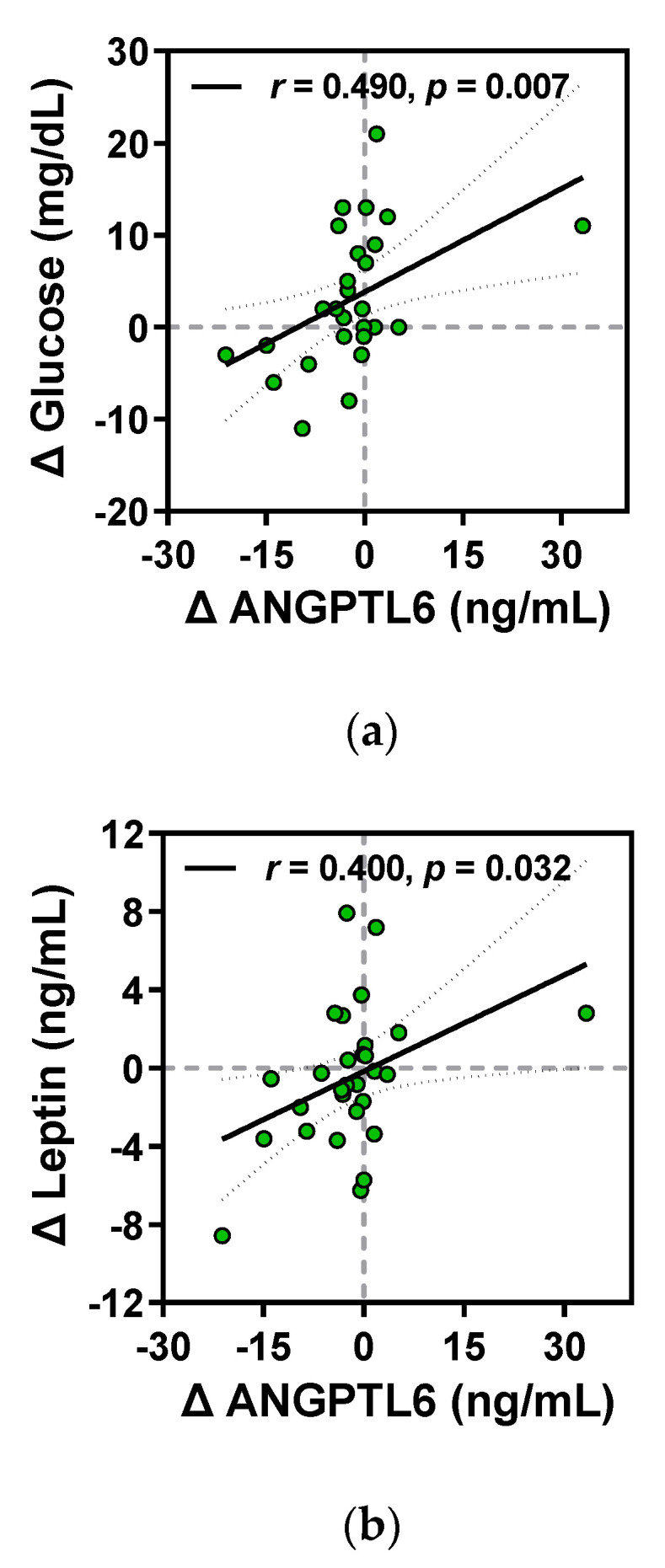
Correlations of Δ ANGPTL6 level with (**a**) Δ glucose and (**b**) Δ leptin levels before and after the intervention. Correlation coefficient (r), corresponding regression and 95% CI lines, and *p*-values are represented in the respective panels. Green-filled circles are individual values.

**Figure 6:**
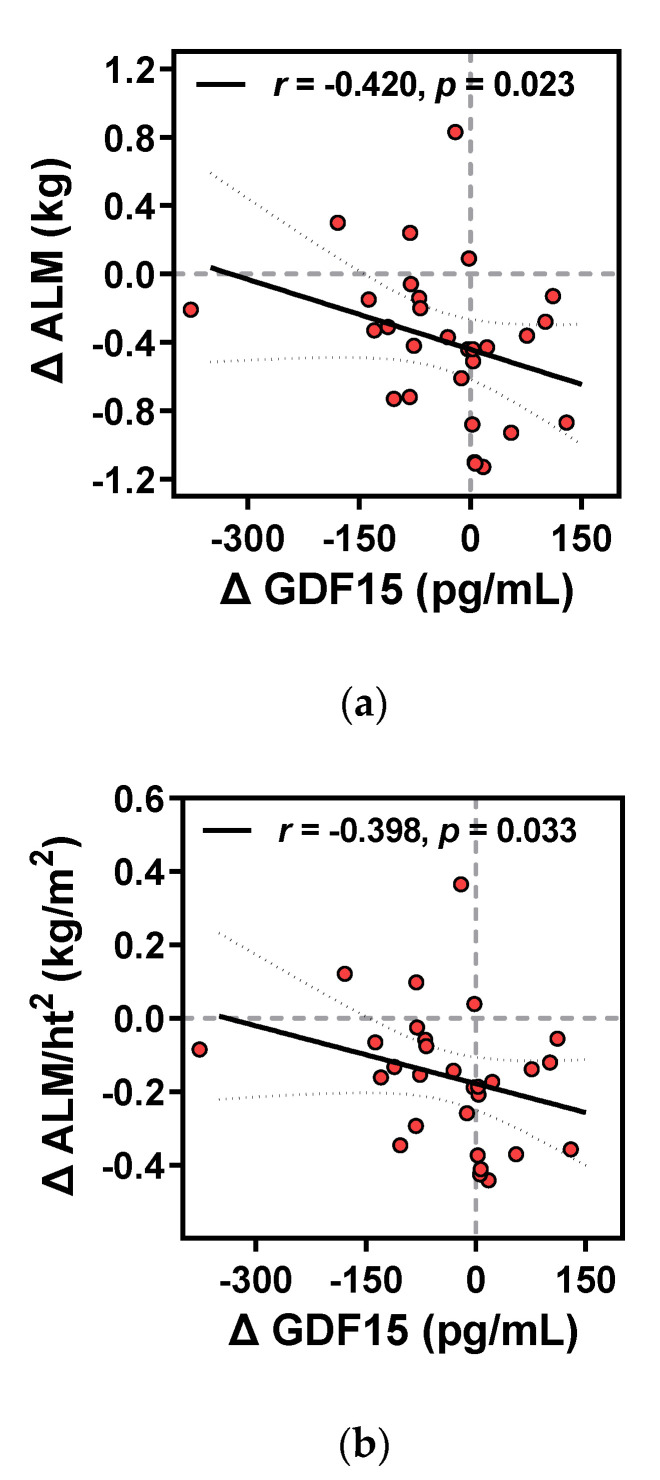
Correlations of Δ GDF15 level with (**a**) Δ appendicular lean mass (ALM) glucose and (**b**) Δ ALM to height ratio (ALM/ht2) before and after the intervention. Correlation coefficient (r), corresponding regression and 95% CI lines, and *p*-values are represented in the respective panels. Red-filled circles are individual values.

The relationship between the intervention-induced changes in primary outcomes and the kinanthropometric and biochemical parameters was determined by Spearman’s correlation analyses using the difference, Δ, of each variable (post-intervention value – baseline value). Significant positive associations were observed between Δ FGF21 and Δ glucose, Δ insulin, Δ HOMA-IR, Δ triglyceride, and Δ leptin (Figure 4). Moreover, a positive relationship existed between Δ ANGPTL6 and Δ glucose and Δ leptin. (Figure 5). Meanwhile, Δ GDF15 levels negatively correlated with only the changes in absolute and relative values of ALM during the intervention period (Figure 6).

## 4. Discussion

To our knowledge, this is the first randomized controlled trial examining the effects of exercise training on serological molecules associated with metabolic disorders. Here, the beneficial effects of exercise on clinical parameters and metabolism-related biomarkers were clearly manifested. Moreover, the changes in the biomarker levels were correlated with the changes in the clinical parameters used to measure cardio-metabolic diseases or age-related diseases risks. These findings may provide novel insights into the understanding of the beneficial effects of exercise on MetS, whose global prevalence can be estimated to be about one-quarter of the world population and has been gradually increasing [27,28].

Aberrant circulating levels of metabolism-related humoral factors indicate metabolic burden and inflexibility. Unresolved metabolic stress may disturb homeostasis and lead to the development and progression of chronic diseases. Thus, our baseline data are consistent with the evidence from previous clinical studies showing that each of the three biomarkers are markedly elevated in MetS patients [17,25,29]. Furthermore, possessing potential as independent predictors of new-onset MetS, these stress markers may be useful in the screening and prediction of individuals at high risk of acquiring metabolic disorders, and in making early intervention decisions during pre-clinical stages [30,31].

Circulating FGF21 is predominantly derived from the liver and produced in the muscles, pancreas, and adipose tissues [32]. It’s signaling is propagated via the activation of the preformed heterodimeric FGF receptors (FGFR) and co-receptor β-klotho complex [33]. Physiologically, FGF21 mainly maintains energy balance via the regulation of gluco-lipid metabolism. FGF21 exerts pleiotropic metabolic benefits through insulin-sensitizing effects, lipolysis promotion, fatty acid oxidation, and/or via stimulating insulin-independent glucose uptake in hepatic and peripheral tissues [34]. Nevertheless, clinical studies have shown that levels of circulating FGF21 are often elevated, notably in pathophysiological states, including T2DM [35], fatty liver diseases [36,37], and coronary heart diseases [38]. Moreover, elevated FGF21 level has recently emerged as an independent predictor for both the prevalence and incidence of MetS, regardless of age, gender, and race/ethnicity [39]. Likewise, habitually active individuals have lower circulating FGF21 concentration alongside better glucose tolerance and insulin sensitivity than the age- and sex-matched sedentary individuals [40]. Like in other hormones associated with obesity-related disorders such as insulin and leptin, these paradoxical discrepancies have led to the interpretation of the elevation of circulating FGF21 in patients with cardio-metabolic diseases as being a compensatory increase or resistance [29,35]. Accordingly, we observed significant correlations of the serum FGF21 level with fasting glucose and insulin levels, and HOMA-IR in a cross-sectional analysis (Appendix A Table A2), consistent with previous findings [29,41]. Given the insulin-mediated induction [41] and the progressive increase of serum FGF21 level with extent of impaired glucose-tolerance [42], these findings imply that insulin resistance is substantially associated with the elevated serum FGF21 concentration or the presence of FGF21 resistance.

Meanwhile, FGF21 has been identified as an exercise-responsive humoral factor in both rodents and humans. Acute and short-term exercises transiently resulted in several fold increase in circulating FGF21 concentration [43,44]. However, the impact of long-term exercise on FGF21 responses varies depending on the participants’ characteristics and exercise protocols [41,45,46]. Here, we found that long-term exercise intervention reduces serum FGF21 levels in MetS patients. This is consistent with the phenomenon previously demonstrated in obese individuals and T2DM patients [45,47], as well as obese mice [48]. Interestingly, the reduction in the FGF21 levels of the study subjects during the intervention period showed positive correlations with the alterations in HOMA-IR as well as in glucose, insulin, and triglyceride levels. The elevation of FGF21 levels in metabolic disorders has been inferred as a compensatory response to attenuate glucose and lipid dyshomeostasis via exerting insulin-sensitizing [49] and triglyceride-lowering effects [50]. Additionally, the expression of FGFRs and β-klotho in metabolic target tissues is notably lower under obesity and T2DM conditions [41] but could be markedly reversed by exercise training in diet-induced obese mice [48]. Thus, our findings imply that aberrant serum FGF21 levels can be reduced by alleviating metabolic stress burden, thereby suggesting that exercise intervention may decrease the compensatory requirement of FGF21 via improving glucose and lipid metabolism in patients with MetS.

GDF15, a divergent member of the transforming growth factor β superfamily, is an emerging biomarker comparable to FGF21, as its circulating levels are noticeably increased in various disease conditions including cancer, metabolic diseases, and aging-related disorders, as well as MetS [51]. In addition, age, smoking, drugs, environmental factors, and prolonged nutritional stressors are other triggers that may alter GDF15 levels [52]. Thus, it has also been referred to as a stress-responsive hormone. Here, we observed that aberrantly elevated serum GDF15 concentrations in MetS have inverse correlations with muscle mass and function as well as a positive correlation with age (Appendix A Table A2). These results agree with the findings of previous cross-sectional studies in elderly women [53] and in patients with non-alcoholic fatty liver disease (the liver manifestation of MetS) [54]. Consistently, basal circulating GDF15 levels were higher in physically inactive patients with impaired mobility causing chronic walking or exercising inabilities. Contrarily, basal circulating GDF15 levels were lower in active cyclists when compared to age-matched control counterparts [55]. Taken together, these findings imply a link between the systemic response to mitochondrial stress and the muscle-mediated metabolic fitness.

As a novel exercise-responsive factor, circulating GDF15 is markedly elevated by both maximal and submaximal acute exercise regimens. However, this transient increase in response to a strenuous bout of physical activity is short-lived, returning to near basal levels within 48 h [55,56]. This phenomenon has been interpreted as an anti-inflammatory response to exercise-induced alterations in pro-inflammatory cytokines, which is similar to that present in patients with low-grade systemic inflammation [55]. Further, training-induced adaptation and its benefits may be attributable to the cumulative effects of each acute exercise bout, but there may be different manifestations of acute response and chronic adaptation to exercise, especially in stress-responsive biomarkers. Although the acute response of GDF15 level has not been investigated, here, we infer that a brief increase in the level of circulating GDF15 may have occurred following exercise sessions, probably contributing to the reinforcement of ISR. Accumulating evidence has shown that exercise training protects against mitochondrial dysfunction and improves mitochondrial biogenesis and respiration [57,58], suggesting the beneficial effects of chronic and prolonged exercise. Consequently, the diminished GDF15 levels in the basal state may be attributable to the mitochondrial flexibility-improving and metabolic stress-relieving actions of cumulative long-term exercise combined with ISR, thereby reflecting alleviated metabolic burden. However, other studies addressing the promotion of weight loss in obese adults and/or elderly individuals have reported that a lifestyle-modifying intervention combining exercise and diet restriction increased the levels of circulating GDF15 [59,60]. These discrepancies can be explained by differences in the study designs and subject characteristics. Moreover, dietary restriction itself is a potent trigger, causing an increase in the levels of circulating GDF15 [52]. Thus, it is difficult to determine whether the upward adaptation of GDF15 release at basal state is mainly attributable to diet or exercise. In this regard, results obtained here, under the conditions of unchanged routine dietary intake, allow us to objectively study the influence of long-term exercise on the basal-state levels of circulating GDF15 in MetS patients.

GDF15 relieves metabolic stress by reducing appetite and enhancing energy expenditure [61,62]. The metabolic effects of GDF15 have recently been revealed by the discovery that glial-derived neurotrophic factor (GDNF) family receptor α-like (GFRAL) binds exclusively to GDF15 with high affinity, and a complex with rearranged during transfection (RET)—a tyrosine kinase co-receptor—is necessary to induce intracellular signaling [61,62]. However, unlike the broad expression of GDF15 in several tissues, GFRAL expression was found to be restricted to the area postrema and nucleus of the solitary tract of the hindbrain [61,62]; thus, the physiological actions of GDF15 on the peripheral tissues, which cannot be explained through GFRAL-mediated anorexigenic effect, have not been clearly elucidated [51]. A recent study showed that human adipose tissue is a possible site of GFRAL expression [62]. Thus, further research on the peripheral tissue cognate receptors of GDF15 is warranted to verify the metabolic benefits accrued by the compensatory/resistive GDF15 increase occurring as an adaptation to exercise training.

As key regulators of MetS pathogenesis, hepatokines have recently emerged as potential biomarkers and therapeutic targets [63]. Among them, ANGPTL6 is regarded as a metabolically beneficial hepatokine since it exerts anti-obesogenic and insulin-sensitizing effects [15]. However, paradoxically, elevated serum ANGPTL6 concentrations have been found prior to MetS onset (in a prospective cohort study) in addition to being found in MetS patients (a cross-sectional study) [17,31]. Hence, we have suggested that circulating ANGPTL6 levels have a strong predictive potential and diagnostic value for MetS. Consistently, our preliminary data confirmed that the levels of circulating ANGPTL6 are characteristically higher in MetS participants than in age-matched healthy volunteers. Moreover, the basal ANGPTL6 level correlates with insulin resistance as well as fasting insulin levels (Appendix A Table A2). As part of a mechanistic study for ANGPTL6 regulation, we have previously found that exercise training lowers serum ANGPTL6 levels in sedentary subjects not having a medical history of metabolic diseases, and its alteration is linked to leptin [64]. However, the reason for the consistent achievement of these exercise training-induced adaptations even in patients with metabolic disorders is still unclear. Thus, our finding that the ANGPTL6 alteration during the exercise training period is notably related to changes in leptin levels even in MetS patients is a notable one in this regard. Hepatic ANGPTL6 expression is regulated by leptin and the exercise training-induced reductions in their serum levels are correlated with each other. Thus, our results strongly indicate that hepatic metabolism can be modulated by exercise-mediated alteration of adipokines, particularly leptin. Also, these findings suggest ANGPTL6 as a potential novel exercise-reducible hepatokine, reflecting the extent of metabolic improvements attributable to exercise training.

There are several limitations to the applications of the findings of the present study. This was a single-center trial involving a relatively small sample size, thereby constraining the generalizability of its results. Further, the short exercise intervention duration and the absence of follow-ups during the detraining period limit our ability to comment on the long-term sustainability of the impressive changes observed in response to exercise. Thus, the individual contribution of each of these exercise modalities in causing the exercise-derived beneficial effects is not clear. The different exercise modalities may be acting synergistically or singly. Therefore, further research determining the individual contributions of different exercise modalities in the alleviation of metabolic burden/disorders via studying their effects on relevant biomolecules (including mitokines and hepatokines) is warranted to develop exercise routines aimed at mitigating metabolic stress conditions.

Exercise prescriptions based on endurance, aerobic interval, and/or muscular-strengthening exercises have been used for improving physical and metabolic parameters in MetS [20,21] as well as prevention [65]. This study showed the effect of a combined exercise modality consisting of endurance and resistance training regimens on the circulating biomarker levels of mitochondrial stress along with improved physical fitness and alleviated cardio-metabolic risks. Therefore, we present additional rationale for exercise training in metabolic syndrome.

## 5. Conclusions

A 12-week combined exercise training attenuated the circulating levels of FGF21, GDF15, and ANGPTL6 and improved physical fitness in MetS patients. Alterations in FGF21 and GDF15 levels were mainly associated with decreased insulin resistance and increased skeletal muscle mass index, respectively. ANGPTL6 exhibited alterations similar to those observed with FGF21, and these alterations were correlated with the changes in leptin levels. Therefore, these findings suggest that the therapeutic application of exercise training can reduce the needs for the compensatory metabolic modulators.

## Figures and Tables

**Table 1:** Changes in kinanthropometric parameters from baseline to 12 weeks.

Variables	Control Group (*n* = 14)		Exercise Group (*n* = 15)	
	Baseline	After 12 Weeks	Baseline	After 12 Weeks
Age (years)	57.5 ± 12.2	–	60.2 ± 7.9	–
Height (cm)	156.3 ± 5.1	–	154.8 ± 5.1	–
Weight (kg)	67.1 ± 11.7	66.3 ± 11.6	63.8 ± 8.6	59.4 ± 8.3
Body mass index (kg/m^2^)	29.5 ± 4.4	29.1 ± 4.3	28.9 ± 3.0	26.7 ± 2.9 *
Waist circumference (cm)	91.7 ± 7.4	89.7 ± 7.5	89.6 ± 5.5	84.1 ± 4.3 *
Body fat (%)	37.6 ± 6.0	38.3 ± 5.8	37.3 ± 5.5	34.9 ± 5.3 *
BFM (kg)	25.6 ± 7.8	25.8 ± 7.7	21.4 ± 5.8	20.9 ± 5.6
ALM (kg)	16.9 ± 0.8	16.3 ± 0.7 *	15.1 ± 0.6	15.5 ± 0.5
ALM/BFM ratio	0.700 ± 0.185	0.669 ± 0.184 *	0.718 ± 0.168	0.753 ± 0.155 *
Systolic blood pressure (mmHg)	132.6 ± 11.4	135.3 ± 14.2	131.3 ± 12.1	129.0 ± 13.5
Diastolic blood pressure (mmHg)	85.3 ± 10.6	85.4 ± 12.6	83.8 ± 10.6	82.3 ± 11.1
Handgrip strength (kg)	27.2 ± 4.8	27.5 ± 4.3	27.3 ± 3.9	28.7 ± 4.2 *
Long jump (cm)	124.7 ± 23.1	118.3 ± 26.0 *	116.1 ± 18.5	117.1 ± 10.4
Sit-up (*n*/30 sec)	11.4 ± 7.7	12.7 ± 7.8	9.3 ± 9.8	13.1 ± 8.3 *
10 m shuttle run (sec)	16.5 ± 1.9	16.4 ± 2.6	16.4 ± 2.1	15.7 ± 1.0
20 m pacer (*n*)	10.2 ± 3.7	10.8 ± 4.4	9.4 ± 3.1	12.5 ± 3.4 *
Sit-and-reach (cm)	13.9 ± 5.0	13.3 ± 3.3	13.9 ± 8.3	16.1 ± 7.1 *

BFM, body fat mass; ALM, appendicular lean mass. Variables are presented as mean ± standard deviation (SD) and compared using the paired *t*-test (* *p* < 0.05 vs. baseline).

**Table 2:** Changes in biochemical profiles and the number of metabolic syndrome components from baseline to 12 weeks in control and exercise groups.

Variables	Control Group (*n* = 14)		Exercise Group (*n* = 15)	
	Baseline	After 12 Weeks	Baseline	After 12 Weeks
Triglyceride (mg/dL)	116.0 (71.0–165.0)	119.0 (87.0–155.0)	139.5 (93.3–188.3)	122.0 (82.8–174.0)
Total cholesterol (mg/dL)	200.6 ± 29.4	195.2 ± 28.3	190.3 ± 26.8	176.5 ± 23.5
HDL-cholesterol (mg/dL)	45.0 (37.0–56.0)	48.5(41.0–53.5)	42.5 (40.0–45.5)	51.0 (40.0–60.0)
LDL-cholesterol (mg/dL)	141.8 ± 22.7	131.3 ± 24.8	130.6 ± 38.7	116.0 ± 39.0
Fasting glucose (mg/dL)	90.5 (85.0–104.8)	95.5 (90.5–105.5)	96.0 (88.0–109.0)	94.0 (89.0–103.0)
Fasting insulin(µIU)	5.80 (3.75–7.53)	6.35 (4.9–11.3)	5.30 (4.50–8.20)	5.20 (4.00–7.50)
HOMA-IR	1.21 (0.86–1.74)	1.62 (1.09–2.83)	1.22 (1.06–1.96)	1.14 (0.92–1.74)
HOMA-β (%)	69.4 (35.1–96.8)	69.2 (45.6–144.9)	56.3 (46.4–79.5)	59.6 (52.4–78.3)
HbA_1c_ (%)	5.55 (5.38–5.73)	5.65 (5.48–5.93)	5.60 (5.40–6.00)	5.50 (5.10–5.90) *
HbA_1c_ (mmol/mol)	37.4 ± 3.6	38.8 ± 3.5	39.3 ± 5.1	37.4 ± 4.9 *
Aspartate aminotransferase (IU/L)	21.5 (16.8–25.3)	24.0 (19.5–26.3)	22.0 (20.0–26.0)	22.0 (18.0–23.0)
Alanine aminotransferase (IU/L)	20.0 (14.5–24.0)	21.0 (18.3–25.8)	18.0 (14.0–22.0)	15.0 (14.0–17.0)
γ-glutamyltransferase (IU/L)	21.0 (15.3–27.3)	27 (16.5–34.5) *	20.0 (11.0–25.0)	16.0 (10.0–23.0)
Highly sensitive C-reactive protein (mg/L)	0.86 ± 0.45	1.49 ± 1.06 *	1.23 ± 2.35	1.02 ± 0.81
Uric acid (mg/dL)	5.10 ± 1.26	4.88 ± 1.32	4.74 ± 1.39	4.45 ± 1.06
Leptin (ng/mL)	10.09 (5.89–17.19)	9.86 (6.89–20.37)	7.83 (4.77–11.92)	7.60 (3.57–9.03) *
Number of components of MetS	4.2 (3.8–4.6)	3.9 (3.3–4.4)	4.1 (3.6–4.6)	3.1 (2.4–3.7) *
0	–	–	–	–
1	–	–	–	1 (6.7)
2	–	1 (7.1)	–	4 (26.7)
3	2 (14.3)	4 (28.6)	5 (33.3)	5 (33.3)
4	7 (50.0)	5 (35.7)	4 (26.7)	3 (20.0)
5	5 (35.7)	4 (28.6)	6 (40.0)	2 (13.3)
^†^*P* value		0.306		0.025

HDL, high-density lipoprotein; LDL, low-density lipoprotein; HOMA-IR and HOMA-β, homeostatic model assessment of insulin resistance and beta cell function. Continuous variables are presented as mean ± SD or median (interquartile range) and compared using paired *t*-test or Wilcoxon signed-ranks test (* *p* < 0.05 vs. baseline). Nominal variables (number of components) are expressed as sample size (proportion) and analyzed by ^†^ Fisher’s exact test.

## Data Availability

The data are not publicly available due to their containing information that could compromise the privacy of research participants.

## Supplementary Materials

The following is available online at https://www.mdpi.com/1660-4601/18/5/2242/s1, Table S1: CONSORT checklist.

## Appendix A

**Table A1:** Baseline demographic and clinical characteristics of the study participants.

Variables	Age-Matched Healthy Control	Metabolic Syndrome Patients	*p*-Value
N	12	30	
Age (year)	58.3 ± 6.0	59.4 ± 10.3	0.541
Body mass index (kg/m^2^)	23.7 ± 2.6	29.2 ± 3.8	0.004
Waist circumference (cm)	78.1 ± 6.0	90.7 ± 9.4	<0.001
Body fat (%)	30.3 ± 7.2	37.4 ± 5.9	0.009
ALM (kg)	16.6 ± 1.8	16.0 ± 2.6	0.442
ALM/ht^2^	6.56 ± 0.36	6.59 ± 0.79	0.876
Handgrip strength (kg)	28.2 ± 4.1	27.3 ± 4.2	0.514
Systolic blood pressure (mmHg)	118.3 ± 10.6	132.4 ± 11.5	<0.001
Diastolic blood pressure (mmHg)	83.4 ± 9.3	84.8 ± 10.5	0.729
Triglyceride (mg/dL)	111.0 (81.5–171.5)	126.0 (86.5–183.0)	0.059
Total cholesterol (mg/dL)	162.3 ± 28.0	195.5 ± 26.3	0.008
HDL-cholesterol (mg/dL)	59.0 (45.0–67.8)	44.5 (40.0–53.0)	0.019
LDL-cholesterol (mg/dL)	141.8 ± 22.7	131.3 ± 24.8	0.265
Fasting glucose (mg/dL)	85.5 (75.5–92.8)	102.2 (98.6–109.4)	0.011
AST (IU/L)	21.0 (16.5–23.8)	17.0 (13.8–23.0)	0.253
ALT (IU/L)	15.0 (10.5–19.8)	22.0 (18.5–25.3)	0.023
γ-GT (IU/L)	13.0 (10.0–17.3)	20.0 (12.8–27.0) *****	0.062

ALM, appendicular lean mass; ALM/ht^2^, appendicular lean mass to height ratio; HDL, high-density lipoprotein; LDL, low-density lipoprotein; AST, aspartate aminotransferase; ALT, alanine aminotransferase; γ-GT, γ-glutamyl transferase. Continuous variables are presented as mean ± SD or median (interquartile range) and compared using independent *t*-test or Mann–Whitney *U* test (* *p* < 0.05 vs. age-matched healthy control).

**Table A2:** Correlation matrix of baseline serum levels of FGF21, GDF15, and ANGPTL6 with parameters evaluated in the study.

Variables		FGF21 (pg/mL)	GDF15 (pg/mL)	ANGPTL6 (ng/mL)	Variables		FGF21 (pg/mL)	GDF15 (pg/mL)	ANGPTL6 (ng/mL)
Age (year)	*r*	−0.067	**0.622**	−0.159	TG (mg/dL)	*r*	**0.525**	0.041	0.276
	*p*	0.729	**<0.001**	0.409		*p*	**0.003**	0.834	0.147
BMI (kg/m^2^)	*r*	−0.014	0.026	0.127	TC (mg/dL)	*r*	0.138	−0.041	0.063
	*p*	0.941	0.893	0.513		*p*	0.477	0.833	0.745
WC (cm)	*r*	0.026	0.008	0.218	HDL-C (mg/dL)	*r*	−0.292	−0.144	−0.268
	*p*	0.893	0.968	0.256		*p*	0.124	0.457	0.159
Body fat (%)	*r*	0.097	0.129	0.087	LDL-C (mg/dL)	*r*	0.12	−0.035	0.072
	*p*	0.616	0.504	0.652		*p*	0.534	0.857	0.709
BFM (kg)	*r*	0.073	0.044	0.186	Glucose (mg/dL)	*r*	**0.386**	−0.043	0.201
	*p*	0.707	0.821	0.333		*p*	**0.039**	0.826	0.296
ALM (kg)	*r*	−0.027	**−0.387**	0.242	Insulin(µIU)	*r*	**0.461**	0.021	**0.464**
	*p*	0.889	**0.038**	0.205		*p*	**0.012**	0.913	**0.011**
ALM/BFM	*r*	−0.069	−0.201	−0.033	HOMA-IR	*r*	**0.509**	0.039	**0.449**
	*p*	0.72	0.295	0.865		*p*	**0.005**	0.841	**0.014**
ALM/ht^2^ (kg/m^2^)	*r*	−0.110	−0.230	0.197	HOMA-β (%)	*r*	0.1	−0.016	0.293
	*p*	0.571	0.23	0.307		*p*	0.606	0.935	0.123
ALM/BMI	*r*	0.042	**−0.424**	0.194	HbA_1c_ (%)	*r*	0.263	0.147	0.107
	*p*	0.827	**0.022**	0.313		*p*	0.168	0.447	0.579
SBP (mmHg)	*r*	0.24	−0.179	0.207	HbA_1c_ (mmol/mol)	*r*	0.259	0.176	0.093
	*p*	0.21	0.352	0.282		*p*	0.174	0.362	0.631
DBP (mmHg)	*r*	0.331	−0.177	0.179	AST (IU/L)	*r*	−0.099	0.136	0.046
	*p*	0.079	0.359	0.353		*p*	0.61	0.483	0.815
HGS (kg)	*r*	−0.132	**−0.478**	−0.098	ALT (IU/L)	*r*	0.218	−0.097	**0.391**
	*p*	0.496	**0.009**	0.614		*p*	0.257	0.615	**0.036**
Long jump (cm)	*r*	−0.226	−0.368	0.059	γ-GT (IU/L)	*r*	0.323	−0.016	−0.101
	*p*	0.299	0.084	0.788		*p*	0.087	0.936	0.602
Sit-up (*n*/30 sec)	*r*	−0.148	−0.258	−0.117	hs-CRP (mg/L)	*r*	0.016	0.051	0.109
	*p*	0.5	0.235	0.595		*p*	0.934	0.793	0.573
10 m shuttle run (sec)	*r*	0.127	0.154	0.114	Uric acid (mg/dL)	*r*	0.251	−0.135	**0.395**
	*p*	0.562	0.483	0.606		*p*	0.188	0.485	**0.034**
20 m pacer (*n*)	*r*	−0.129	−0.324	−0.087	Leptin (ng/mL)	*r*	0.033	−0.013	0.266
	*p*	0.558	0.132	0.693		*p*	0.863	0.947	0.164

FGF 21, fibroblast growth factor 21; GDF15, growth differentiation factor 15; ANGPTL6, angiopoietin-like 6; BMI, body mass index; WC, waist circumference; BFM, body fat mass; ALM, appendicular lean mass; ALM/ht^2^; appendicular lean mass to height ratio; ALM/BMI, appendicular lean mass to body mass index ratio; SBP, systolic blood pressure; DBP, diastolic blood pressure; HGS, handgrip strength; TG, triglyceride; TC, total cholesterol; HDL-C, high-density lipoprotein cholesterol; LDL-C, low-density lipoprotein cholesterol; HOMA-IR, homeostatic model assessment of insulin resistance; HOMA-β, homeostatic model assessment of β cell function; HbA_1c_, glycosylated hemoglobin; AST, aspartate aminotransferase; ALT, alanine aminotransferase; γ-GT, γ-glutamyl transferase; hs-CRP, high-sensitivity C-reactive protein. Bolded values denote statistical significance at *p* < 0.05.

## References

[1] Low S., Khoo K.C.J., Wang J., Irwan B., Sum C.F., Subramaniam T., Lim S.C., Wong T.K.M. (2019). Development of a metabolic syndrome severity score and its association with incident diabetes in an Asian population-results from a longitudinal cohort in Singapore. Endocrine.

[2] Mottillo S., Filion K.B., Genest J., Joseph L., Pilote L., Poirier P., Rinfret S., Schiffrin E.L., Eisenberg M.J. (2010). The metabolic syndrome and cardiovascular risk a systematic review and meta-analysis. J. Am. Coll. Cardiol..

[3] DeBoer M.D., Filipp S.L., Gurka M.J. (2018). Use of a Metabolic Syndrome Severity Z Score to Track Risk During Treatment of Prediabetes: An Analysis of the Diabetes Prevention Program. Diabetes Care.

[4] Alberti K.G., Zimmet P., Shaw J., IDF Epidemiology Task Force Consensus Group (2005). The metabolic syndrome—A new worldwide definition. Lancet.

[5] James A.M., Collins Y., Logan A., Murphy M.P. (2012). Mitochondrial oxidative stress and the metabolic syndrome. Trends Endocrinol. Metab..

[6] Bhatti J.S., Bhatti G.K., Reddy P.H. (2017). Mitochondrial dysfunction and oxidative stress in metabolic disorders—A step towards mitochondria based therapeutic strategies. Biochim. Biophys. Acta Mol. Basis Dis..

[7] Alemany M. (2011). Utilization of dietary glucose in the metabolic syndrome. Nutr. Metab..

[8] Thyfault J.P., Krogh-Madsen R. (2011). Metabolic disruptions induced by reduced ambulatory activity in free-living humans. J. Appl. Physiol..

[9] Li H., Zhang J., Jia W. (2013). Fibroblast growth factor 21: A novel metabolic regulator from pharmacology to physiology. Front. Med..

[10] Morovat A., Weerasinghe G., Nesbitt V., Hofer M., Agnew T., Quaghebeur G., Sergeant K., Fratter C., Guha N., Mirzazadeh M. (2017). Use of FGF-21 as a Biomarker of Mitochondrial Disease in Clinical Practice. J. Clin. Med..

[11] Chung H.K., Ryu D., Kim K.S., Chang J.Y., Kim Y.K., Yi H.S., Kang S.G., Choi M.J., Lee S.E., Jung S.B. (2017). Growth differentiation factor 15 is a myomitokine governing systemic energy homeostasis. J. Cell. Biol..

[12] Boenzi S., Diodato D. (2018). Biomarkers for mitochondrial energy metabolism diseases. Essays Biochem..

[13] Kim S.H., Kim K.H., Kim H.K., Kim M.J., Back S.H., Konishi M., Itoh N., Lee M.S. (2015). Fibroblast growth factor 21 participates in adaptation to endoplasmic reticulum stress and attenuates obesity-induced hepatic metabolic stress. Diabetologia.

[14] Fujita Y., Taniguchi Y., Shinkai S., Tanaka M., Ito M. (2016). Secreted growth differentiation factor 15 as a potential biomarker for mitochondrial dysfunctions in aging and age-related disorders. Geriatr. Gerontol. Int..

[15] Oike Y., Akao M., Yasunaga K., Yamauchi T., Morisada T., Ito Y., Urano T., Kimura Y., Kubota Y., Maekawa H. (2005). Angiopoietin-related growth factor antagonizes obesity and insulin resistance. Nat. Med..

[16] Ebert T., Kralisch S., Loessner U., Jessnitzer B., Stumvoll M., Fasshauer M., Tonjes A. (2014). Relationship between serum levels of angiopoietin-related growth factor and metabolic risk factors. Horm. Metab. Res..

[17] Namkung J., Koh S.B., Kong I.D., Choi J.W., Yeh B.I. (2011). Serum levels of angiopoietin-related growth factor are increased in metabolic syndrome. Metabolism.

[18] McGee S.L., Hargreaves M. (2020). Exercise adaptations: Molecular mechanisms and potential targets for therapeutic benefit. Nat. Rev. Endocrinol..

[19] Durstine J.L., Moore G.E., Painter P.L. (2016). American College of Sports Medicine. ACSM’s Exercise Management for Persons with Chronic Diseases and Disabilities.

[20] Stensvold D., Tjonna A.E., Skaug E.A., Aspenes S., Stolen T., Wisloff U., Slordahl S.A. (2010). Strength training versus aerobic interval training to modify risk factors of metabolic syndrome. J. Appl. Physiol..

[21] Amanat S., Sinaei E., Panji M., MohammadporHodki R., Bagheri-Hosseinabadi Z., Asadimehr H., Fararouei M., Dianatinasab A. (2020). A Randomized Controlled Trial on the Effects of 12 Weeks of Aerobic, Resistance, and Combined Exercises Training on the Serum Levels of Nesfatin-1, Irisin-1 and HOMA-IR. Front. Physiol..

[22] Colado J.C., Garcia-Masso X., Triplett T.N., Flandez J., Borreani S., Tella V. (2012). Concurrent validation of the OMNI-resistance exercise scale of perceived exertion with Thera-band resistance bands. J. Strength Cond. Res..

[23] Haff G., Triplett N.T. (2016). National Strength & Conditioning Association (U.S.) Essentials of Strength Training and Conditioning.

[24] Matthews D.R., Hosker J.P., Rudenski A.S., Naylor B.A., Treacher D.F., Turner R.C. (1985). Homeostasis model assessment: Insulin resistance and beta-cell function from fasting plasma glucose and insulin concentrations in man. Diabetologia.

[25] Schindler K., Vila G., Hoppichler F., Lechleitner M., Luger A., Anderwald C., Hoefler J., Tomasec G., Kautzky-Willer A., Ludvik B. (2012). The impact of type 2 diabetes on circulating adipokines in patients with metabolic syndrome. Obes. Facts.

[26] Park B.S., Yoon J.S. (2013). Relative skeletal muscle mass is associated with development of metabolic syndrome. Diabetes Metab. J..

[27] Saklayen M.G. (2018). The Global Epidemic of the Metabolic Syndrome. Curr. Hypertens. Rep..

[28] Lee S.E., Han K., Kang Y.M., Kim S.O., Cho Y.K., Ko K.S., Park J.Y., Lee K.U., Koh E.H., Taskforce Team of Diabetes Fact Sheet of the Korean Diabetes, A (2018). Trends in the prevalence of metabolic syndrome and its components in South Korea: Findings from the Korean National Health Insurance Service Database (2009–2013). PLoS ONE.

[29] Zhang X., Yeung D.C., Karpisek M., Stejskal D., Zhou Z.G., Liu F., Wong R.L., Chow W.S., Tso A.W., Lam K.S. (2008). Serum FGF21 levels are increased in obesity and are independently associated with the metabolic syndrome in humans. Diabetes.

[30] Choi J.R., Kim J.Y., Park I.H., Huh J.H., Kim K.W., Cha S.K., Park K.S., Sohn J.H., Park J.T., Koh S.B. (2018). Serum Fibroblast Growth Factor 21 and New-Onset Metabolic Syndrome: KoGES-ARIRANG Study. Yonsei Med. J..

[31] Namkung J., Sohn J.H., Chang J.S., Park S.W., Kim J.Y., Koh S.B., Kong I.D., Park K.S. (2019). Increased Serum Angiopoietin-Like 6 Ahead of Metabolic Syndrome in a Prospective Cohort Study. Diabetes Metab. J..

[32] Itoh N. (2014). FGF21 as a Hepatokine, Adipokine, and Myokine in Metabolism and Diseases. Front. Endocrinol..

[33] Kharitonenkov A., Dunbar J.D., Bina H.A., Bright S., Moyers J.S., Zhang C., Ding L., Micanovic R., Mehrbod S.F., Knierman M.D. (2008). FGF-21/FGF-21 receptor interaction and activation is determined by betaKlotho. J. Cell. Physiol..

[34] Staiger H., Keuper M., Berti L., Hrabe de Angelis M., Haring H.U. (2017). Fibroblast Growth Factor 21-Metabolic Role in Mice and Men. Endocr. Rev..

[35] Chavez A.O., Molina-Carrion M., Abdul-Ghani M.A., Folli F., Defronzo R.A., Tripathy D. (2009). Circulating fibroblast growth factor-21 is elevated in impaired glucose tolerance and type 2 diabetes and correlates with muscle and hepatic insulin resistance. Diabetes Care.

[36] Li H., Fang Q., Gao F., Fan J., Zhou J., Wang X., Zhang H., Pan X., Bao Y., Xiang K. (2010). Fibroblast growth factor 21 levels are increased in nonalcoholic fatty liver disease patients and are correlated with hepatic triglyceride. J. Hepatol..

[37] Shen J., Chan H.L., Wong G.L., Choi P.C., Chan A.W., Chan H.Y., Chim A.M., Yeung D.K., Chan F.K., Woo J. (2012). Non-invasive diagnosis of non-alcoholic steatohepatitis by combined serum biomarkers. J. Hepatol..

[38] Lin Z., Wu Z., Yin X., Liu Y., Yan X., Lin S., Xiao J., Wang X., Feng W., Li X. (2010). Serum levels of FGF-21 are increased in coronary heart disease patients and are independently associated with adverse lipid profile. PLoS ONE.

[39] Ong K.L., McClelland R.L., Allison M.A., Kokkinos J., Wu B.J., Barter P.J., Rye K.A. (2019). Association of elevated circulating fibroblast growth factor 21 levels with prevalent and incident metabolic syndrome: The Multi-Ethnic Study of Atherosclerosis. Atherosclerosis.

[40] Lee S.Y., Burns S.F., Ng K.K.C., Stensel D.J., Zhong L., Tan F.H.Y., Chia K.L., Fam K.D., Yap M.M.C., Yeo K.P. (2020). Fibroblast Growth Factor 21 Mediates the Associations between Exercise, Aging, and Glucose Regulation. Med. Sci. Sports Exerc..

[41] Kruse R., Vienberg S.G., Vind B.F., Andersen B., Hojlund K. (2017). Effects of insulin and exercise training on FGF21, its receptors and target genes in obesity and type 2 diabetes. Diabetologia.

[42] Lin Z., Gong Q., Wu C., Yu J., Lu T., Pan X., Lin S., Li X. (2012). Dynamic change of serum FGF21 levels in response to glucose challenge in human. J. Clin. Endocrinol. Metab..

[43] Kim K.H., Kim S.H., Min Y.K., Yang H.M., Lee J.B., Lee M.S. (2013). Acute exercise induces FGF21 expression in mice and in healthy humans. PLoS ONE.

[44] Cuevas-Ramos D., Almeda-Valdes P., Meza-Arana C.E., Brito-Cordova G., Gomez-Perez F.J., Mehta R., Oseguera-Moguel J., Aguilar-Salinas C.A. (2012). Exercise increases serum fibroblast growth factor 21 (FGF21) levels. PLoS ONE.

[45] Yang S.J., Hong H.C., Choi H.Y., Yoo H.J., Cho G.J., Hwang T.G., Baik S.H., Choi D.S., Kim S.M., Choi K.M. (2011). Effects of a three-month combined exercise programme on fibroblast growth factor 21 and fetuin-A levels and arterial stiffness in obese women. Clin. Endocrinol..

[46] Banitalebi E., Kazemi A., Faramarzi M., Nasiri S., Haghighi M.M. (2019). Effects of sprint interval or combined aerobic and resistance training on myokines in overweight women with type 2 diabetes: A randomized controlled trial. Life Sci..

[47] Shabkhiz F., Khalafi M., Rosenkranz S., Karimi P., Moghadami K. (2020). Resistance training attenuates circulating FGF-21 and myostatin and improves insulin resistance in elderly men with and without type 2 diabetes mellitus: A randomized controlled clinical trial. Eur. J. Sport. Sci..

[48] Geng L., Liao B., Jin L., Huang Z., Triggle C.R., Ding H., Zhang J., Huang Y., Lin Z., Xu A. (2019). Exercise Alleviates Obesity-Induced Metabolic Dysfunction via Enhancing FGF21 Sensitivity in Adipose Tissues. Cell. Rep..

[49] Mashili F.L., Austin R.L., Deshmukh A.S., Fritz T., Caidahl K., Bergdahl K., Zierath J.R., Chibalin A.V., Moller D.E., Kharitonenkov A. (2011). Direct effects of FGF21 on glucose uptake in human skeletal muscle: Implications for type 2 diabetes and obesity. Diabetes Metab. Res. Rev..

[50] Schlein C., Talukdar S., Heine M., Fischer A.W., Krott L.M., Nilsson S.K., Brenner M.B., Heeren J., Scheja L. (2016). FGF21 Lowers Plasma Triglycerides by Accelerating Lipoprotein Catabolism in White and Brown Adipose Tissues. Cell. Metab..

[51] Chang J.Y., Hong H.J., Kang S.G., Kim J.T., Zhang B.Y., Shong M. (2020). The Role of Growth Differentiation Factor 15 in Energy Metabolism. Diabetes Metab. J..

[52] Patel S., Alvarez-Guaita A., Melvin A., Rimmington D., Dattilo A., Miedzybrodzka E.L., Cimino I., Maurin A.C., Roberts G.P., Meek C.L. (2019). GDF15 Provides an Endocrine Signal of Nutritional Stress in Mice and Humans. Cell. Metab..

[53] Hofmann M., Halper B., Oesen S., Franzke B., Stuparits P., Tschan H., Bachl N., Strasser E.M., Quittan M., Ploder M. (2015). Serum concentrations of insulin-like growth factor-1, members of the TGF-beta superfamily and follistatin do not reflect different stages of dynapenia and sarcopenia in elderly women. Exp. Gerontol..

[54] Koo B.K., Um S.H., Seo D.S., Joo S.K., Bae J.M., Park J.H., Chang M.S., Kim J.H., Lee J., Jeong W.I. (2018). Growth differentiation factor 15 predicts advanced fibrosis in biopsy-proven non-alcoholic fatty liver disease. Liver Int..

[55] Conte M., Martucci M., Mosconi G., Chiariello A., Cappuccilli M., Totti V., Santoro A., Franceschi C., Salvioli S. (2020). GDF15 Plasma Level Is Inversely Associated With Level of Physical Activity and Correlates With Markers of Inflammation and Muscle Weakness. Front. Immunol..

[56] Kleinert M., Clemmensen C., Sjoberg K.A., Carl C.S., Jeppesen J.F., Wojtaszewski J.F.P., Kiens B., Richter E.A. (2018). Exercise increases circulating GDF15 in humans. Mol. Metab..

[57] Joseph A.M., Adhihetty P.J., Leeuwenburgh C. (2016). Beneficial effects of exercise on age-related mitochondrial dysfunction and oxidative stress in skeletal muscle. J. Physiol..

[58] Tsai H.H., Chang S.C., Chou C.H., Weng T.P., Hsu C.C., Wang J.S. (2016). Exercise Training Alleviates Hypoxia-induced Mitochondrial Dysfunction in the Lymphocytes of Sedentary Males. Sci. Rep..

[59] Laurens C., Parmar A., Murphy E., Carper D., Lair B., Maes P., Vion J., Boulet N., Fontaine C., Marques M. (2020). Growth and differentiation factor 15 is secreted by skeletal muscle during exercise and promotes lipolysis in humans. JCI Insight.

[60] Zhang H., Fealy C.E., Kirwan J.P. (2019). Exercise training promotes a GDF15-associated reduction in fat mass in older adults with obesity. Am. J. Physiol. Endocrinol. Metab..

[61] Emmerson P.J., Wang F., Du Y., Liu Q., Pickard R.T., Gonciarz M.D., Coskun T., Hamang M.J., Sindelar D.K., Ballman K.K. (2017). The metabolic effects of GDF15 are mediated by the orphan receptor GFRAL. Nat. Med..

[62] Mullican S.E., Lin-Schmidt X., Chin C.N., Chavez J.A., Furman J.L., Armstrong A.A., Beck S.C., South V.J., Dinh T.Q., Cash-Mason T.D. (2017). GFRAL is the receptor for GDF15 and the ligand promotes weight loss in mice and nonhuman primates. Nat. Med..

[63] Esfahani M., Baranchi M., Goodarzi M.T. (2019). The implication of hepatokines in metabolic syndrome. Diabetes Metab. Syndr..

[64] Kim M.J., Namkung J., Chang J.S., Kim S.J., Park K.S., Kong I.D. (2018). Leptin regulates the expression of angiopoietin-like 6. Biochem. Biophys. Res. Commun..

[65] Tjonna A.E., Ramos J.S., Pressler A., Halle M., Jungbluth K., Ermacora E., Salvesen O., Rodrigues J., Bueno C.R., Munk P.S. (2018). EX-MET study: Exercise in prevention on of metabolic syndrome—A randomized multicenter trial: Rational and design. BMC Public Health.

